# A Key Role of CD8+ T Cells in Controlling of Tuberculosis Infection

**DOI:** 10.3390/diagnostics13182961

**Published:** 2023-09-15

**Authors:** Igor Kudryavtsev, Yulia Zinchenko, Maria Serebriakova, Tatiana Akisheva, Artem Rubinstein, Andrei Savchenko, Alexandr Borisov, Vasilij Belenjuk, Anna Malkova, Piotr Yablonskiy, Dmitry Kudlay, Anna Starshinova

**Affiliations:** 1 Institution of Experimental Medicine, Department of Immunology, 197376 St-Petersburg, Russia; igorek1981@yandex.ru (I.K.); m-serebryakova@yandex.ru (M.S.); akisheva@yandex.ru (T.A.); arrubin6@mail.ru (A.R.); 2 Almazov National Medical Research Centre, 197341 St-Petersburg, Russia; 3 Research Institute of Phthisiopulmonology, 191036 St-Petersburg, Russia; ulia-zinchenko@yandex.ru (Y.Z.); piotr_yablonskii@mail.ru (P.Y.); 4 Federal Research Center «Krasnoyarsk Science Center» of the Siberian Branch of the Russian Academy of Sciences, Scientific Research Institute of Medical Problems of the North, 660022 Krasnoyarsk, Russia; aasavchenko@yandex.ru (A.S.); 2410454@mail.ru (A.B.); dyh.88@mail.ru (V.B.); 5 Department of Molecular Biology, Faculty of Natural Sciences, Ariel University, Ariel 40700, Israel; anya.malkova.95@mail.ru; 6 St. Petersburg Research Institute of Phthisiopulmonology, Ligovskii Prospect, 2–4, 191036 St-Petersburg, Russia; 7 Department of Pharmacology, I.M. Sechenov First Moscow State Medical University, 119991 Moscow, Russia; d624254@gmail.com; 8 NRC Institute of Immunology FMBA of Russia, 115552 Moscow, Russia

**Keywords:** CD3+CD8+, tuberculosis, Tc1, Tc17, Tc2, chemokine receptors, memory CD8+ T cells

## Abstract

The main role in the control of tuberculosis infection is played by macrophages and Th1 and CD8+ T cells. The study aimed to identify the most diagnostically significant CD8+ T cell subsets in tuberculosis patients. Methods: Peripheral blood samples from patients with clinical, radiological, and bacteriologically confirmed pulmonary tuberculosis (TB, n = 32) and healthy subjects (HC, n = 31) were collected and analyzed using 10-color flow cytometry. Results: The frequency of the EM4 CD3+CD8+ cells was reduced in the peripheral blood of patients with pulmonary tuberculosis, while the relative and absolute number of EM1 CD3+CD8+ cells increased compared to the control group. CD57 expression was reduced in patients with pulmonary tuberculosis on EM1, EM2, and pE1 CD3+CD8+ cells, whereas the EM3 cells had a high level of CD57 expression. The relative and absolute number of Tc2 (CCR6–CXCR3–) cells in peripheral blood in patients with pulmonary tuberculosis was increased, while the frequency of Tc1 (CCR6–CXCR3+) was decreased, compared to healthy donors. Conclusions: Patients with pulmonary tuberculosis have an abnormal CD3+CD8+ cell profile and demonstrate their impaired maturation and functional activity.

## 1. Introduction

Tuberculosis (TB) remains a deadly infectious disease that causes a large number of deaths worldwide [1]. At the UN Assembly in 2021, the World Health Organization’s report on the global fight against TB declared that the COVID-19 pandemic had severely undermined the gains made against the disease worldwide, with TB mortality rates rising for the first time in more than a decade [2].

According to the WHO, after the COVID-19 pandemic, there was an increase in mortality due to the disease from 1.4 million TB deaths in 2019 to 1.5 million in 2021. At the same time, there has been an 18% decrease in the number of reported new tuberculosis (TB) cases from 7.1 million in 2019 to 5.8 million in 2020 [2]. At the same time, analyses have shown that global TB notification rates are expected in 40% of children and 21% of adults [3,4], highlighting the need to focus maximum attention on the issues of early detection.

Before the COVID-19 tuberculosis pandemic, a quarter of the world’s population was estimated by the WHO to be infected with *M. tuberculosis*. At the same time, 4% of those infected with *M. tuberculosis* with latent TB infection may develop active TB under the influence of various factors [5,6,7].

It is known that the presence of concomitant pathology, immunosuppression, HIV infection, malnutrition, and diabetes mellitus are the main risk factors for the development of tuberculosis infection [2,8,9]. The emergence of COVID-19 has led to adjustments being made, as it may be one of the factors influencing the development of active tuberculosis due to changes in immune status against the background of infection with the SARS-CoV-2 virus [10]. Therefore, obtaining new data on changes in immunologic parameters in tuberculosis patients will be of high importance in determining the prognosis of the course of the disease in the future.

Despite the existence of a large number of studies on the immune status of tuberculosis patients, there are certain gaps in understanding the interaction of *M. tuberculosis* with a host organism. Currently, it remains unclear what immunological processes lead to the activation, progression, and generalization of tuberculosis infection [11,12].

It is known that the immune response in tuberculosis is cell-mediated [13,14]. More than 65% of the total number of lymphocytes are T cells, which are the most important human immune effector cells. T cells mainly include helper or inducible T cells (CD3+CD8-CD4+), cytotoxic T cells (CD3+CD8+CD4-), and NKT cells [15,16]. Moreover, it is the protective cellular immunity against tuberculosis that is mainly mediated by CD4+ T-lymphocytes but supported by CD8+ T-lymphocytes [17]. However, the role of CD8+ T-lymphocytes in the development and course of tuberculosis infection has not been clearly defined to date, which is the purpose of our study. The study aims to identify the most diagnostically significant CD8+T-lymphocyte subpopulations in tuberculosis patients.

## 2. Materials and Methods

### 2.1. Patients

A prospective study from 2017 to 2021, analyzing the results of an immunological study of the peripheral blood of patients with pulmonary tuberculosis (n = 32, main group), aged 18 to 65 years in comparison with a group of healthy subjects (n = 31, control group), was conducted. Inclusion criteria: presence of clinical, radiological, and bacteriologically confirmed pulmonary tuberculosis. Exclusion criteria: Presence of immunosuppression, HIV infection, cancer, autoimmune pathology, pregnancy, breastfeeding, alcoholism, drug addiction, and chronic pathology in the exacerbation stage. The control group included healthy individuals not in contact with tuberculosis patients, without chronic pathology, with no changes in chest radiography, and with negative results on immunodiagnostics the immunologic test (Diaskintest^®^, Generium, Russia). We also want to mention that acute COVID-19 and COVID-19 convalescents were excluded from the TB and healthy control groups.

The characteristics of the tuberculosis patients are presented in Table 1.

The diagnosis of pulmonary tuberculosis was verified through the detection of *M. tuberculosis* in sputum and/or MBT DNA according to molecular-genetic and bacteriological methods, with the presence of typical changes according to radiation examination.

### 2.2. Sample Collection

Peripheral blood samples from the patients were collected before treatment initiation. Five milliliters of peripheral blood was collected from each TB patient and healthy subject using K3EDTA anticoagulant tubes. Collected peripheral blood samples were processed immediately. CD8+ T cell subset immunophenotyping was performed within several hours (less than 6 h at 20–22 °C) after blood collection.

### 2.3. Immunophenotyping of Peripheral Blood CD8+ T Cell Subset Maturation Stages and CD57 Expression

For the immunophenotyping of CD8+ T cell subset maturation stages, 200 μL of whole peripheral blood samples was stained with mouse anti-human monoclonal antibodies that recognized cell-surface human CD57, CD62L, CD28, CD27, CD4, CD8, CD3, CD45RA, and CD45. The detailed characteristics of antibodies are given in Supplementary Table S1. All antibodies were manufactured by Beckman Coulter Inc. (Indianapolis, IN, USA), and were used according to the manufacturer’s recommendations. Next, all samples were incubated at room temperature in the dark for 15 min, followed by red blood cell lysis for 15 min in the dark using 2 mL of VersaLyse Lysing Solution (Beckman Coulter, Inc., USA) supplied with 50 μL of IOTest 3 Fixative Solution (Beckman Coulter, Inc., USA). Finally, 200 μL of Flow-Count Fluorospheres (Beckman Coulter, Indianapolis, IN, USA) was added, and sample acquisition was performed using a 3/10 Navios flow cytometer (Beckman Coulter, Indianapolis, IN, USA). At least 20,000 CD8+ T cells were analyzed in each sample. The gating strategy for the CD8+ T cell subset maturation stages is shown in Supplementary Figure S1 and has been described in detail previously [18].

### 2.4. Immunophenotyping of Peripheral Blood ‘Polarized’ CD8+ T Cell Subsets

For the immunophenotyping of ‘polarized’ CD8+ T cell subsets, 200 μL of whole peripheral blood samples was stained with the following mouse anti-human monoclonal antibodies, which interacted with human CD45RA (Beckman Coulter, Indianapolis, IN, USA), human CD62L (Beckman Coulter, Indianapolis, IN, USA), human CXCR5 (CD185, BioLegend, Inc., San Diego, CA, USA), human CCR6 (BioLegend, Inc., San Diego, CA, USA), human CXCR3 (CD183, BioLegend, Inc., San Diego, CA, USA), human CD3 (Beckman Coulter, Indianapolis, IN, USA), human CD8 (BioLegend, Inc., San Diego, CA, USA), and human CCR4 (CD194, BioLegend, Inc., San Diego, CA USA). The detailed characteristics of antibodies are given in Supplementary Table S2. All antibodies were used according to the manufacturer’s recommendations. Blood samples were stained with antibodies at room temperature for 15 min in the dark. Then, red blood cells were lysed by adding 2 mL of Versa Lyse Lysing Solution (Beckman Coulter, Inc., Indianapolis, IN, USA) with 25 µL of IOTest 3 Fixative Solution (Beckman Coulter, Inc., Indianapolis, IN, USA) in the dark at room temperature for 15 min. Next, all samples were washed (330× *g* for 8 min) twice with sterile PBS supplemented with 2% of fetal calf serum (FCS) (Sigma–Aldrich Co., Saint Louis, MO, USA), resuspended in 500 µL of fresh PBS with 2% neutral formalin (Sigma–Aldrich Co., Saint Louis, MO, USA), and subjected to a flow cytometry analysis. At least 20,000 CD3+CD8+ cells were collected from each sample. The gating strategy for the major ‘polarized’ CD8+ T cell subsets is shown in Supplementary Figure S2 and has been described in detail previously [18].

### 2.5. Statistical Analysis

The flow cytometry data were analyzed using Kaluza software v2.3 (Beckman Coulter, Indianapolis, IN, USA). Statistical analysis was performed with Statistica 7.0 (StatSoft, Tulsa, OK, USA) and GraphPad Prism 8 (GraphPad Software Inc., San Diego, CA, USA) software packages. Normality was checked using Pearson’s chi-squared test. Flow cytometry data were presented as a percentage of cells, and the absolute number of CD8+ T cell subsets was presented as the number of cells per 1 μL of whole peripheral blood. All data were presented as the median and interquartile range, Me (Q25; Q75). The differences between patients with pulmonary tuberculosis and the healthy controls were analyzed using a nonparametric Mann–Whitney U-test with a *p* < 0.05 value.

## 3. Results

### 3.1. Main Peripheral Blood T Cell Subsets in Patients with Pulmonary Tuberculosis

**Figure 1:**
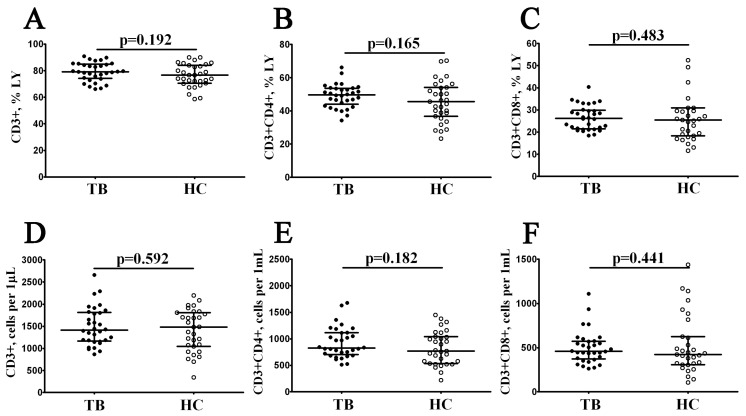
Comparison of relative and absolute numbers of main circulating T cell subsets in patients with pulmonary tuberculosis. Scatter plots (**A**–**F**) showing the percentages (the percentage of T cell subset within the total lymphocyte subset, LY) and absolute numbers (number of cells per 1 μL of whole peripheral blood) of T cells (CD3+), T-helpers (Th, CD3+CD4+), and CD8+ T cells (Tcyt, CD3+CD8+), respectively. Black circles denote patients with pulmonary tuberculosis (TB, n = 32); white circles—the healthy control (HC, n = 31).

Surface expression of CD3 was used to define the total T cell subset in patients with pulmonary tuberculosis (Figure 1), and we found no significant differences in the relative and absolute numbers of CD3+ T cells between the TB and HC groups (79.23% (74.31; 84.95) vs. 76.72% (70.60; 84.08) and 1415 cells/1 μL (1170; 1813) vs. 1481 cells/1 μL (1044; 1811) with *p* = 0.191 and *p* = 0.591, respectively). Notably, we also did not find changes in CD3+CD4+ and CD3+CD8+ frequencies between groups (Figure 1).

### 3.2. Alterations in Peripheral Blood CD8+ T Cell Maturation Subsets in Patients with Pulmonary Tuberculosis

**Figure 2:**
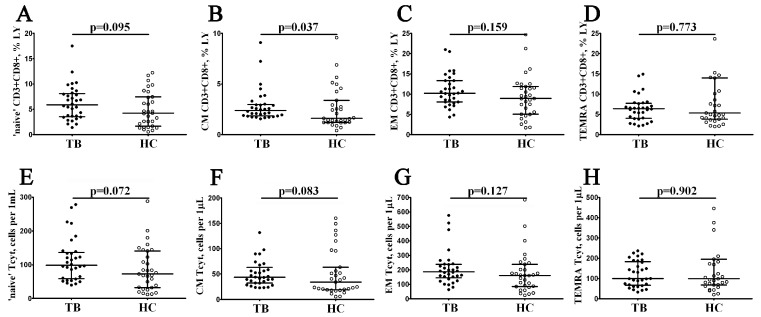
Relative and absolute frequencies of peripheral blood CD8+ T cell subsets with different patterns of CD45RA and CD62L expression in patients with pulmonary tuberculosis. Scatter plots (**A**–**H**), showing the percentages and absolute numbers of ‘naïve’ (CD45RA+CD62L+), central memory (CM, CD45RA−CD62L+), effector memory (EM, CD45RA−CD62L−), and terminally differentiated CD45RA-positive effector memory (TEMRA, CD45RA+CD62L−) CD8+ T cells, respectively. Black circles denote patients with pulmonary tuberculosis (TB, n = 32); white circles—the healthy control (HC, n = 31).

We next focused on enumerating the different maturation CD8+ T cell subsets identified using a variable expression of CD45RA and CD62L. We identified ‘naïve’ (CD45RA+CD62L+), central memory (CM, CD45RA–CD62L+), effector memory (EM, CD45RA–CD62L–), and terminal effector CD45RA-positive effector memory (TEMRA, CD45RA+CD62L–) CD8+ T cells. We noticed that the relative number of CM CD8+ T cells was significantly increased in patients with pulmonary tuberculosis (2.34% (1.87; 2.98) vs. 1.62% (1.22; 3.38) with *p* = 0.037, Figure 2B).

### 3.3. Alterations in EM and TEMRA CD8+ T Cell Subsets in Patients with Pulmonary Tuberculosis

**Figure 3:**
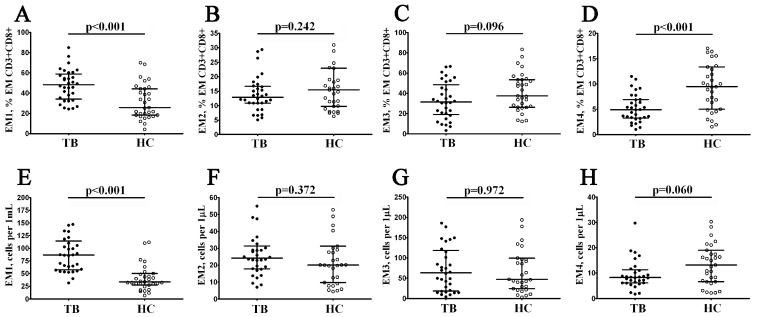
Alterations in relative and absolute number of EM CD8+ T cell subsets with different patterns of CD27 and CD28 expression in patients with pulmonary tuberculosis. Scatter plots (**A**–**H**)—EM CD8+ T cell were subdivided into EM1 (CD27+CD28+), EM2 (CD27+CD28−), EM3 (CD27−CD28−), and EM4 (CD27−CD28+) subsets, respectively. Scatter plots (**A**–**D**)—the relative numbers of EM1, EM2, EM3, and EM4 cells within the EM CD8+ T cell subset; scatter plots (**E**–**H**)—EM1, EM2, EM3, and EM4 cell concentrations (number of cells per 1μL of peripheral whole blood). Black circles denote patients with pulmonary tuberculosis (TB, n = 32); white circles—the healthy control (HC, n = 31).

Co-stimulatory CD27 and CD28 play essential functions in T cell activation during specific antigen recognition, presented by antigen-presenting cells [19] and constitutively expressed on ‘naïve’ and central memory CD8+ T cells. The total EM cells’ expression of these two co-stimulatory molecules allows us to define the four distinct subsets, including the EM1 (CD27+CD28+), EM2 (CD27+CD28−), EM3 (CD27–CD28−), and EM4 (CD27–CD28+) subsets, as has been shown previously by Romero et al. [20]. We observed that, compared with HD, TB patients had increased frequencies of most immature EM1 cells (48.27% (34.08; 58.81) vs. 25.78% (18.33; 44.34) and 87 cells/1 μL (58; 114) vs. 34 cells/1 μL (27; 50) with *p* < 0.001 in both cases, Figure 3A,E). We also noticed that the relative numbers of EM4 CD8+ T cells were decreased in patients with tuberculosis compared to the healthy controls (4.92% (3.28; 6.92) vs. 9.48% (5.03; 13.35), *p* < 0.001, Figure 3D).

**Figure 4:**
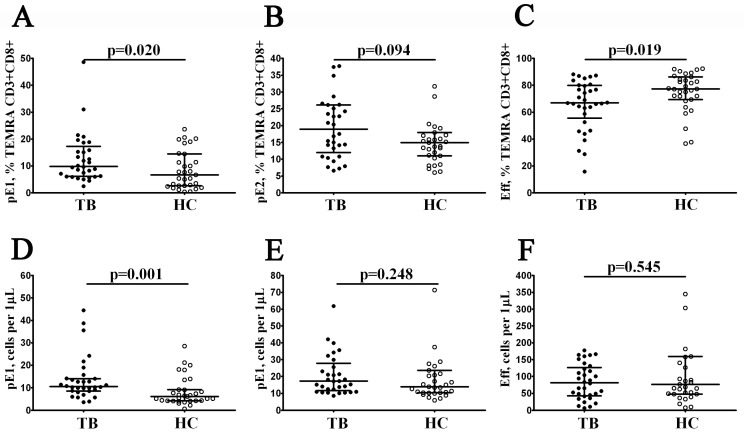
Alterations in the relative and absolute number of TEMRA CD8+ T cell subsets with different patterns of CD27 and CD28 expression in patients with pulmonary tuberculosis. Scatter plots (**A**–**F**)—TEMRA CD8+ T cells were subdivided into CD27+CD28+ pE1, CD27+CD28− pE2, and CD27–CD28− E subsets, respectively. Scatter plots (**A**–**C**)—the relative numbers of pE1, pE2, and effector cells within the TEMRA CD8+ T cell subset; scatter plots (**D**–**F**)—pE1, pE2, and effector cell concentrations (number of cells per 1 μL of peripheral whole blood). Black circles denote patients with pulmonary tuberculosis (TB, n = 32); white circles—the healthy control (HC, n = 31).

Moreover, TEMRA CD8+ T cells also expressed different patterns of CD27 and CD28 and could be subdivided into pre-effector type 1 cells (pE1, CD27+CD28+), pre-effector type 2 cells (pE2, CD27+CD28−), and effector cells (Eff, CD27–CD28−), as suggested by Rufer et al. [21]. We noticed that the levels of immature pE1 cells were increased in patients with tuberculosis compared to the healthy controls (9.86% (6.17; 17.28) vs. 6.63% (2.66; 14.45) and 11 cells/1 μL (9; 14) vs. 6 cells/1 μL (4; 9) with *p* = 0.020 and *p* = 0.001, respectively; Figure 4A,D). Furthermore, the related frequency of the effector CD27–CD28− CD8+ t cells was decreased in TB patients vs. the control group (66.90% (55.64; 79.83) vs. 77.33% (69.46; 86.21) with *p* = 0.019, Figure 4C).

Thus, our results demonstrate that EM and TEMRA CD8+ T cell subsets were enriched with immature EM1 and EM4, as well as pE1 cells, respectively, while EM4 and most mature cells—effector subset within TEMRA cells—were decreased.

### 3.4. Alterations in CD57 Expression on CD8+ T Cell Subsets from Patients with Pulmonary Tuberculosis

Next, we analyzed CD57 expression using CD8+ T cells. Previously, it was demonstrated that CD57 is a marker of highly differentiated mature CD8+ T cells that are needed to control intracellular pathogens, including CMV and other viruses in humans [22,23]. Furthermore, CD57 cell-surface expression was closely linked with intracellular granzyme A, granzyme B, and perforin accumulation [24]. We found that several CD8+ T cell subsets from patients with tuberculosis, including EM1, EM2, and pE1 cells showed decreased levels of CD57+ cells, while EM3 cells were enriched with CD57+ cells compared to healthy controls (Table 2). Our data indicated alterations in the effector capacities of CD8+ T cells during *M. tuberculosis* infection. Interestingly, we found no significant differences in the absolute numbers of CD57-positive cells in diverse CD8+ T cell subsets (Supplementary Table S3).

### 3.5. Imbalance in Peripheral Blood Main ‘Polarized’ CD8+ T Cell Subsets in Patients with Pulmonary Tuberculosis

**Figure 5:**
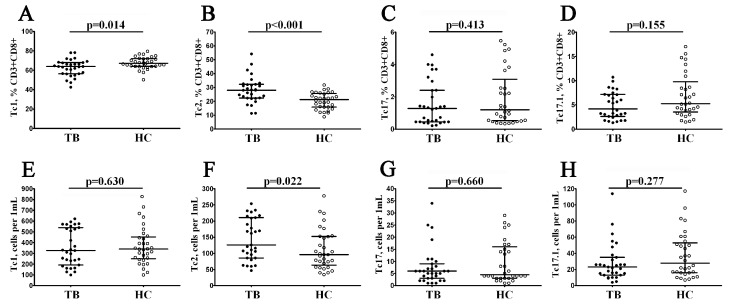
Imbalance in relative and absolute numbers of peripheral blood Tc1, Tc2, and Tc17 cells and double-positive Tc17.1 cells in patients with pulmonary tuberculosis. Scatter plots (**A**–**H**)—Tc1 (CCR6−CXCR3+), Tc2 (CCR6−CXCR3−), Tc17 (CCR6+CXCR3−), and double-positive Tc17.1 (CCR6+CXCR3+) frequencies, respectively. Scatter plots (**A**–**D**)—the relative numbers of Tc1, Tc2, Tc17, and Tc17.1 within the total CD8+ T cell subset; scatter plots (**E**–**H**)—Tc1, Tc2, Tc17, and Tc17.1 concentrations (number of cells per 1 μL of peripheral whole blood). Black circles denote patients with pulmonary tuberculosis (TB, n = 32); white circles—the healthy control (HC, n = 31).

To assess the potential contribution of different ‘polarized’ CD8+ T cell subsets in tuberculosis pathogenesis, we further analyzed the peripheral blood CD8+ T cells for inflammatory subsets classified by CCR6 and CXCR3 co-expression. Thus, we identified four main CD8+ T cell subsets in our blood samples, including Tc1 (CCR6–CXCR3+), Tc2 (CCR6–CXCR3–), Tc17 (CCR6+CXCR3–), and double-positive Tc17.1 (CCR6+CXCR3+), as has been proposed previously [25,26]. We found that the relative numbers of CCR6–CXCR3+ Tc1 within total CD8+ T cells were decreased in TB patients vs. the control group (63.91% (56.46; 67.99) vs. 67.16% (64.07; 71.95) with *p* = 0.014, Figure 5A). Furthermore, patients with tuberculosis also increased Tc2 frequencies (both in relative and absolute numbers), compared to the healthy controls (28.03% (22.31; 32.33) vs. 21.27% (15.87; 25.66) and 126 cells/1 μL (86; 211) vs. 96 cells/1 μL (63; 152) with *p* < 0.001 and *p* = 0.022, respectively, Figure 5B,F). Thus, the ‘polarized’ CD8+ T cell subsets’ imbalance indicated changes in the immune function of CD8+ T cells during *M. tuberculosis* infection.

### 3.6. Imbalance in Tc1, Tc2, and Tc17 Cells in Peripheral Blood CD8+ T Cell Maturation Subsets in Patients with Pulmonary Tuberculosis

**Figure 6:**
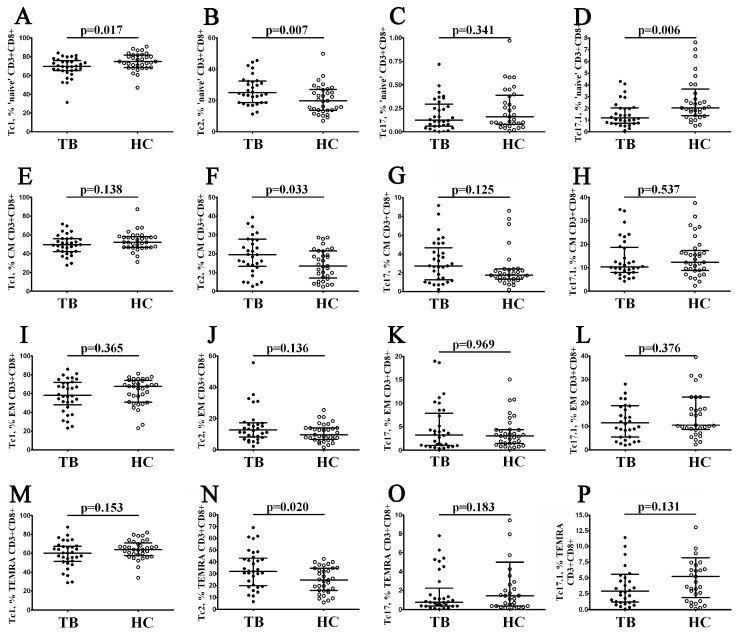
Imbalance in ‘polarized’ Tc1, Tc2, Tc17, and Tc17.1 cells in main peripheral blood maturation CD8+ T cell subsets in patients with pulmonary tuberculosis. Scatter plots (**A**–**P**) show the percentages of Tc1, Tc2, Tc17, and Tc17.1 cells within ‘naïve’ (CD45RA+CD62L+), central memory (CM, CD45RA−CD62L+), effector memory (EM, CD45RA−CD62L–), and terminally differentiated CD45RA-positive effector memory (TEMRA, CD45RA+CD62L) CD8+ T cells, respectively. Black circles denote patients with pulmonary tuberculosis (TB, n = 32); white circles—healthy control (HC, n = 31).

Finally, we analyzed the frequencies of inflammatory CD8+ T cell subsets in different maturation stages of CD8+ T cells. Thus, we first analyzed the frequency of Tc1, Tc2, Tc17, and Tc17.1 cells within the ‘naïve’ CD8+ T cells that were generated in the thymus and were not activated by their corresponding antigens. We found that, in TB patients, the related numbers on Tc1 and Tc17.1 were decreased vs. the control group (69;77% (65.14; 75.68) vs. 74.96% (68.55; 81.60) and 1.19% (0.75; 2.05) vs. 2.05% (1.40; 3.28) with *p* = 0.017 and *p* = 0.006, respectively, Figure 6A,D), while the frequency of Tc2 was significantly increased (25.04% (18.74; 32.49) vs. 19.92% (13.87; 26.78) with *p* = 0.006, Figure 6B). We also noticed elevated levels of Tc2 cells in TB patients within central memory CD8+ T cells compared to healthy controls (19.49% (13.30; 27.71) vs. 13.40% (7.51; 21.36) with *p* = 0.033, Figure 6F). Finally, increased levels of Tc2 cells were identified within TEMRA CD8+ T cells in patients with tuberculosis vs. the healthy control group (32.10% (19.87; 43.06) vs. 24.66% (15.73; 34.56) with *p* = 0.020, Figure 6N). Thus, we noted markedly elevated proportions of peripheral blood Tc2 cells (lacking cytotoxic properties, but capable of Th2 cytokines secretion) on different maturation stages of CD8+ T cells in patients with *M. tuberculosis* infection.

## 4. Discussion

The main role in the local immunological response against *M. tuberculosis* is played by macrophages and Th1 [27]. These cells are the main participants in the delayed-type hypersensitivity reaction and contribute to limiting the spread of mycobacterial infection [28]. However, Th2, when producing IL-4, suppresses the Th1 response and worsens the course of the infection, contributing to its spread [27]. Reports have shown an increase in Th2 cells in patients with cavitary tuberculosis [29]. Cytokines released by these CD4+ T cells induce alternative macrophage activation and collagen deposition at the site of inflammation [27]. This process is observed in common forms of tuberculosis. Therefore, in patients with pulmonary tuberculosis, the balance of Th1/Th2 is very important, as it determines the further course of this infection.

*M. tuberculosis* uses multiple immune evasion strategies and can also effectively modulate adaptive immune responses via the inhibition of different T cell functional activities [30]. It was found that *M. tuberculosis* was able suppress T cells by stimulating mesenchymal stem cells, recruiting them to the site of infection; meanwhile, mesenchymal stem cells produced different mediators that down-regulated effector T cell functions [31]. Furthermore, *M. tuberculosis*-specific CD4+CD25+FoxP3+ T cells isolated from the blood and pleural fluid were capable of suppressing IFN-g production in TB patients that potently restricted protective immune responses during tuberculosis by suppressing the effector T cell responses against the bacterium in the site of infection [32]. Finally, *M. tuberculosis* antigens could chronically stimulate antigen-specific CD4+ T cells, making ESAT-6-specific T cells functionally exhausted due to chronic antigenic stimulation [33].

We found no differences in the CD3+CD8+ frequencies between patients with pulmonary tuberculosis and healthy controls. Similarly, Ocaña-Guzmán et al. found that the frequency of CD8+ T cell distribution was similar in HC and TB groups [34]. On the contrary, Chávez-Galán et al. found that TB patients had a high frequency of CD8+ cells in peripheral blood [35]. Absolute counts of CD8+ T cells decreased significantly with an increase in the number of pulmonary lobes involved, and the peripheral blood CD8+ T cells’ concentration had the most impact on the presence or absence of cavities [36]. Moreover, Chen et al. noticed that patients with active pulmonary tuberculosis had fewer CD8+ T cells compared to the healthy control and patients with a latent *M. tuberculosis* infection [37]. Furthermore, in patients with active pulmonary tuberculosis, CD8+ T cell counts decreased and were negatively associated with the extent of the lesions detected in the chest using computed tomography; however, they increased significantly after 4 weeks of antituberculosis treatment [38].

We described an abnormal CD8+ T cell profile in patients with pulmonary tuberculosis, which resulted in imbalanced distribution of CD8+ maturation and ‘polarized’ subsets. We found that EM and TEMRA CD8+ T cell subsets were enriched with immature EM1 and pE1 cells, respectively, while effector CD8+ cells were decreased. Previously, Ocaña-Guzmán et al. observed a decrease in the levels of the ‘naïve’ CD8+ T cell subset and an increase in the EM cells in groups of patients infected with *M. tuberculosis* [34]. But, the expression of granzyme A, granzyme B, and perforin in patients with active pulmonary tuberculosis was higher than those in the healthy controls and patients with latent *M. tuberculosis* infection, and it decreased after curing [37].

Interestingly, CD57 was previously proposed as a cell molecule of end-stage, senescent CD8+ T cells in HIV patients exhibiting highly cytotoxic potential, while exhausted CD8+ T cells tended to have a low expression of CD57 [39]. Furthermore, exhausted CD57+ CD8+ T cells had a low proliferative capacity [40]. Thus, the decreased levels of CD57 in immature EM1, EM4, and pE1 (expressing high levels of CD27 and CD28) cells could be linked with their ineffective function activities, including altered clonal expansion in response to antigen stimulation or even the slow homeostatic turnover of memory CD8+ T cells and the altered formation of effector CD8+ T cells in general. Recently, Chen et al. found that CD8+ T cells from patients with tuberculosis exhibited a high expression of LAG-3 that inhibited the proliferation and maturation of these cells, and elevated LAG-3 expression correlated with the functional defects of CD8+ T cells and TB severity [41]. Moreover, CD8+CD28− T cells in the peripheral blood of patients with pulmonary tuberculosis were significantly higher than those in the healthy control group, and the increase in atypical CD8+CD28− T cells was related to the progression of *M. tuberculosis* infection [42]. Finally, the CD8+ T cells of patients with tuberculosis presented a decrease in the frequency of CD8+ cells expressing activating receptor NKG2D, but the frequencies of the CD8+ T cells that co-expressed the NKG2A inhibitor receptor were similar to the control group [35]. Thus, all these data point to an imbalance in the effector and regulatory mechanisms of CD8+ T cells, which compromised cell function and could promote pathogen immune escape during *M. tuberculosis* infection.

We noted that, in peripheral blood samples from patients with *M. tuberculosis* infection, markedly decreased levels of CXCR3+ Tc1 cells are known to produce high levels of IFNγ and TNFα, as well as granzyme and perforin, which contribute to the killing of yeast-infected host cells [43]. Similarly, CXCR3+CD8+ T cells were depleted from the circulation in TB, and this CD8+ T cell subset slightly and progressively recovered by the end of the anti-tuberculosis therapy [34]. Furthermore, the levels of CXCR3 ligands—chemokine MIG, IP-10, and I-TAC—in plasma samples from patients with active pulmonary tuberculosis were discovered to be higher than those in patients with latent *M. tuberculosis* infection and cured pulmonary tuberculosis patients [37]. Moreover, the pleural fluid concentration of MIG, IP-10, and I-TAC from patients with active pulmonary tuberculosis increased significantly compared to their plasma levels. Thus, CXCR3 and its ligands could play an important part in the regulation of Tc1 CD8+ T cell migration to the site of infection. Immunohistochemically stained lung biopsies show a large number of CD8+ T cells in tuberculosis granulomas [27].

It was shown that the effector subsets of CD8+ T cells can migrate to sites of inflammation, perform cytolytic functions, and produce effector cytokines [44]. Moreover, effector perforin- and granulysin-expressing CD8+ T cells were detected in tuberculous granulomas in patients with pulmonary tuberculosis [45]. Thus, CD8+ T cells expressed effector molecules and infiltrated pulmonary tissue. Furthermore, effector CD8+ T cells upregulated CXCR3 expression on their cell membranes following infection with a variety of intracellular pathogens [46]. Hickman et al. reported that CXCR3 could affect not only CD8+ T cell migration into sites of infection but also migration within inflamed tissues, as CD8+ T cells locate pathogen-infected targets for killing [47]. Therefore, a decrease in the peripheral blood of Tc1 and the most mature effector subset within TEMRA cells may indicate their migration to the site of the tuberculosis infection.

However, these cytotoxic cells have a reduced expression of perforin, granzyme, and granulizing, which indicates the functional disorders of these cells during tuberculosis infection. CD8+ T cells have a peripheral localization in granulomas, while CD4+ T cells occupy their center [27,45]. *M. tuberculosis* is located in the center of the granuloma. Therefore, IFN-γ-producing CD4+ T cells are the most involved in the anti-tuberculosis response, and CD8+ T cells are not in close contact with *M. tuberculosis*-infected cells, which impairs pathogen clearance by these cytotoxic lymphocytes.

We also noticed increased levels of Tc2 cells in peripheral blood samples from patients with tuberculosis. It was shown that Tc2 cells have reduced cytotoxic activity compared to Tc1 cells [48]. However, Tc2 cells have a similar cytokine profile to Th2 cells that can suppress Th1 and Tc1 effector functions [49]. Therefore, an increase in the peripheral blood Tc2 cells in patients with tuberculosis can be closely linked with the low effectiveness of the type 1 immune response. Furthermore, type 2 immune response dominance may lead to disease progression, since enhanced Th2 polarization was one of the consequences of the progressive TB disease [50]. Interestingly, significantly augmented levels of the IL-17+CD8+ T cells were detected in patients with TB infection compared to the healthy group, suggesting a potentially deleterious role of Tc17 CD8+ T cells during active pulmonary tuberculosis [51]. Previously, it was shown that circulating CD8+ T cells from TB patients had increased the levels of the activation marker HLA-DR as well as chemokine receptors (CCR2, CCR3, and CXCR4) [51].

It is necessary to study further changes in the level of CD8+ T cells in tuberculosis patients after COVID-19 infection, as well as in the post-COVID-19 syndrome. As the results of past studies have shown, COVID-19 patients have certain immunogenetic features that determine the severity of COVID-19 infection and the consequences of post-COVID-19 infection [52]. Also, in patients with tuberculosis after COVID-19, there were disorders of immune response with changes in the level of naive T cells, and their ratio with B-cells changed in patients who underwent COVID-19 [10,53].

Our research had some limitations. We did not assess the functional capacity of CD8+ T cell subsets, and we also did not analyze serum cytokines levels. Moreover, we did not conduct immunohistochemical studies of the affected lung tissues.

## 5. Conclusions

Thus, the obtained results and literary data suggest that CD8+ T cells play an important role in controlling *M. tuberculosis* infection. We are sure that the determination of the subpopulation of CD8+ T cells can further help in predicting the course of tuberculosis.

## Figures and Tables

**Table 1:** Demographic, clinical, and bacteriological characteristics of Tbc patients.

Characteristics	Tbc Patients n (%) (n = 32)
Men	22 (68.7)
Women	10 (31.3)
Age	36.5 (±10.6) years
Clinical symptoms	29 (90.6)
Fever	20/32 (62.5)
General weakness	21/32 (65.6)
Sweating	18/32 (56.2)
Weight loss	21/32 (65.6)
Respiratory symptoms	
Cough	22/32 (68.7)
Shortness of breath	11/32 (34.3)
Chest pain	5/32 (15.6)
X-Ray and CT changes	
Infiltrates in the lungs	15 (46.8)
Focus on the lungs	11 (34.3)
Focal infiltrates and focus in the lungs	6 (18.7)
Bacteriologic data	
Sputum positive for MBT	32 (100.0)
MDR	10 (31.2)

**Table 2:** CD57 expression on diverse CD8+ T cell subsets in patients with pulmonary tuberculosis.

CD8+ T Cell Subset	Phenotype	TB Group (n = 32) (%, Med Q25; Q75)	Healthy Control (n = 31) (%, Med Q25; Q75)	Significant Differences (*p* < 0.05)
Naïve	CD45RA+CD62L+	0.96 (0.48; 3.21)	1.62 (0.63; 3.49)	0.417
CM	CD45RA–CD62L+	3.56 (1.75; 7.86)	3.01 (1.65; 5.28)	0.343
EM:	CD45RA–CD62L–	36.97 (29.85; 44.67)	39.87 (30.94; 59.8)	0.211
EM1	CD27+CD28+	5.63 (3.49; 8.46)	10.60 (6.57; 12.82)	0.001
EM2	CD27+CD28−	37.59 (27.69; 49.63)	46.43 (34.60; 62.03)	0.035
EM3	CD27–CD28−	81.96 (76.81; 90.52)	76.75 (66.55; 83.60)	0.037
EM4	CD27–CD28+	10.25 (4.98;16.80)	12.51 (8.40; 20.78)	0.114
TEMRA:	CD45RA+CD62L–	59.83 (51.15; 70.09)	64.42 (53.26; 76.4)	0.157
pE1	CD27+CD28+	3.53 (2.34; 6.37)	6.55 (2.87; 10.00)	0.029
pE2	CD27+CD28−	34.19 (27.25; 42.69)	44.31 (32.08; 52.00)	0.243
effectors	CD27–CD28−	74.44 (68.59; 84.89)	80.08 (65.07; 86.90)	0.433

## Data Availability

Availability of data and materials. All source data are in the supplementary files to the article, if you need clarifications, or need additional information, you can write to the email: starshinova_777@mail.ru.

## Supplementary Materials

The following supporting information can be downloaded at: https://www.mdpi.com/article/10.3390/diagnostics13182961/s1, Table S1. List of monoclonal antibodies for immunophenotyping of peripheral blood CD8+ T cell subset maturation stages and CD57 expression (all antibodies were manufactured by Beckman Coulter, Indianapolis, IN, USA). Table S2. List of monoclonal antibodies for immunophenotyping of peripheral blood ‘polarized’ CD8+ T cell subsets (CD25, CD4, CD8 were manufactured by Beckman Coulter, Indianapolis, IN, USA, and CD183, CD185, CD194, CD196, CD3, CD197, CD45RA were manufactured by BioLegend, Inc., San Diego, CA, USA). Table S3. Absolute numbers CD57-expressing cells in diverse CD8+ T cell subsets in patients with pulmonary tuberculosis. Figure S1. Flow cytometry immunophenotyping gating strategy for CD8+ T cell subset maturation stages and assessing CD57 expression (shown in dot plots). (A) Total lymphocyte subset purification based on side scatter and bright CD45 expression; (B) artifact exclusion included time gating; (C) doublets exclusion from the analysis by using the ratio between integral and peak forward scatter signals; (D) discrimination between lymphocytes and cell debris; (E) total CD3 expression-based T cell subset gaiting; (F) CD8+ T cells detected within total CD3+ T cell population; (G) CD45RA and CD62L co-expression in identifying the four major CD8+ T cell maturation subsets: ‘naïve’ CD45RA+CD62L+ (naïve), central memory CD45RA-CD62L+ (CM), effector memory CD45RA−CD62L− (EM), and terminally differentiated CD45RA-positive effector memory CD45RA+CD62L− (TEMRA) CD8+ T cells; (H) EM1 (CD27+CD28+), EM2 (CD27+CD28−), EM3 (CD27−CD28−), and EM4 (CD27−CD28+) subsets were identified within total effector memory CD45RA−CD62L− CD8+ T cells; (I) differentiation of terminally differentiated CD8+ T cells (TEMRA) by assessing CD27 and CD28 expression allowed us to subdivide CD8+ T cells into pre-effector type 1 cells (pE1, CD27+CD28+), pre-effector type 2 cells (pE2, CD27+CD28−), and effector cells (E, CD27−CD28−); (J–L) CD57 expression by total CD8+ T cell population, naïve, and TEMRA CD8+ T cell subsets, respectively. Figure S2. Gating and analysis strategy for ‘polarized’ CD8+ T cell subsets immunophenotyping using flow cytometry. Dot plot (A)—artifact exclusion included time gating; dot plot (B)—doublets exclusion from the analysis using the ratio between integral and peak forward scatter signals; dot plot (C)—total lymphocyte subset purification based on side scatter and forward scatter; dot plot (D)—total T cell subset gaiting based on CD3 expression; dot plot (E)—detection of CD8+ T cells within total CD3+ T cell subset; dot plot (F)—identification of four main CD8+ T cell maturation subsets: ‘naïve’, central memory (CM), effector memory (EM), and TEMRA CD8+ T cells; dot plots (G–L)—examples of CXCR5, CCR4, CXCR3, and CCR6 expression by total CD8+ T cells; dot plots–examples of Tc1 (CCR6−CXCR3+), Tc2 (CCR6−CXCR3−), Tc17 (CCR6+CXCR3−), and double-positive Tc17.1 (CCR6+CXCR3+) detection within total CD8+ T cell subset and TEMRA CD8+ T cell, respectively.
