# Supplementary material for: A Key Role of CD8+ T Cells in Controlling of Tuberculosis Infection
Source: Diagnostics (Basel). 2023 Sep 15;13(18):2961. doi: 10.3390/diagnostics13182961 (PMC10528134; doi:10.3390/diagnostics13182961)
Supplement: Supplementary file 1 [file diagnostics-13-02961-s001.zip › diagnostics-2569841-supplementary.pdf]

Supplementary file.

Supplementary Table S1. List of monoclonal antibodies for immunophenotyping of peripheral blood CD8+ T cell subset maturation stages and CD57 expression (all antibodies were manufactured by Beckman Coulter, Indianapolis, IN, USA).

N	Antigen	Fluorochome	Clone	Isotype	Cat. number
**1**	CD57	FITC	NC1	IgM, mouse	B49188
**2**	CD56	PE	N901 (NKH-1)	IgG1, mouse	A07788
**3**	CD62L	ECD	DREG56	IgG1, mouse	IM2713 U
**4**	CD28	PC5.5	CD28.2	IgG1, mouse	B24027
**5**	CD27	PC7	1A4CD27	IgG1, mouse	A54823
**6**	CD4	APC	13B8.2	IgG1, mouse	IM2468
**7**	CD8	APC-AF700	B9.11	IgG1, mouse	B76279
**8**	CD3	APC-AF750	UCHT1	IgG1, mouse	A94680
**9**	CD45RA	Pacific Blue	2H4	IgG1, mouse	A82946
**10**	CD45	Krome Orange	J33	IgG1, mouse	B36294

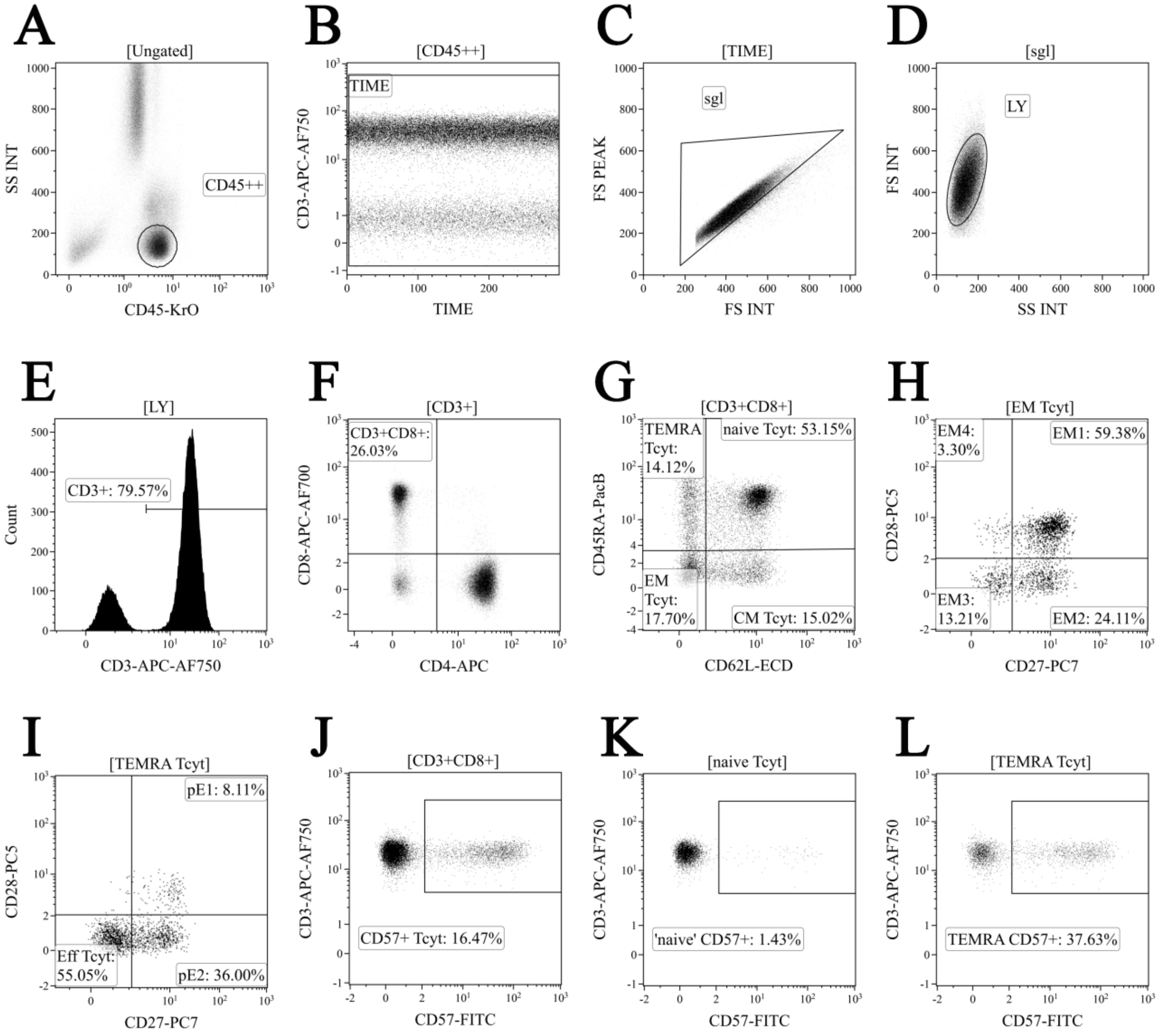

Supplementary Figure S1. Flow cytometry immunophenotyping gating strategy for CD8+ T cell subset maturation stages and assessing CD57 expression (shown in dot plots). (A) Total lymphocyte subset purification based on side scatter and bright CD45 expression; (B) artifact exclusion included time gating; (C) doublets exclusion from the analysis by using the ratio between integral and peak forward scatter signals; (D) discrimination between lymphocytes and cell debris; (E) total CD3 expression-based T cell subset gaiting; (F) CD8+ T cells detected within total CD3+ T cell population; (G) CD45RA and CD62L co-expression in identifying the four major CD8+ T cell maturation subsets: 'naïve' CD45RA+CD62L+ (naïve), central memory CD45RA-CD62L+ (CM), effector memory CD45RA−CD62L− (EM), and terminally differentiated CD45RA-positive effector memory CD45RA+CD62L− (TEMRA) CD8+ T cells; (H) EM1 (CD27+CD28+), EM2 (CD27+CD28−), EM3 (CD27−CD28−), and EM4 (CD27−CD28+) subsets were identified within total effector memory CD45RA−CD62L− CD8+ T cells; (I) differentiation of terminally differentiated CD8+ T cells (TEMRA) by assessing CD27 and CD28 expression allowed us to subdivide CD8+ T cells into pre-effector type 1 cells (pE1, CD27+CD28+), pre-effector type 2 cells (pE2, CD27+CD28-), and effector cells (E, CD27−CD28−); (J–L) CD57 expression by total CD8+ T cell population, naïve, and TEMRA CD8+ T cell subsets, respectively.

Supplementary Table S2. List of monoclonal antibodies for immunophenotyping of peripheral blood 'polarized' CD8+ T cell subsets (CD25, CD4, CD8 were manufactured by Beckman Coulter, Indianapolis, IN, USA, and CD183, CD185, CD194, CD196, CD3, CD197, CD45RA were manufactured by BioLegend, Inc., San Diego, CA, USA).

N	Antigen	Fluorochome	Clone	Isotype	Cat. number
**1**	CD183 (CXCR3)	Alexa Fluor 488	G025H7	Mouse IgG1, k	353710
**2**	CD25	PE	B1.49.9	IgG2a Mouse	A07774
**3**	CD185 (CXCR5)	PE/Dazzle™ 594	J252D4	Mouse IgG1, k	356928
**4**	CD194 (CCR4)	PerCP/Cy5.5	L291H4	Mouse IgG1, k	359406
**5**	CD196 (CCR6)	PE/Cy7	G034E3	Mouse IgG2b, k	353418
**6**	CD4	APC	13B8.2	IgG1 Mouse	IM2468
**7**	CD8	APC-AF700	B9.11	IgG1 Mouse	B49181
**8**	CD3	APC/Cy7	HIT3a	Mouse IgG2a, k	300318
**9**	CD197 (CCR7)	Brilliant Violet 421	G043H7	Mouse IgG2a, k	353208
**10**	CD45RA	Brilliant Violet 510	HI100	Mouse IgG2b, k	304142

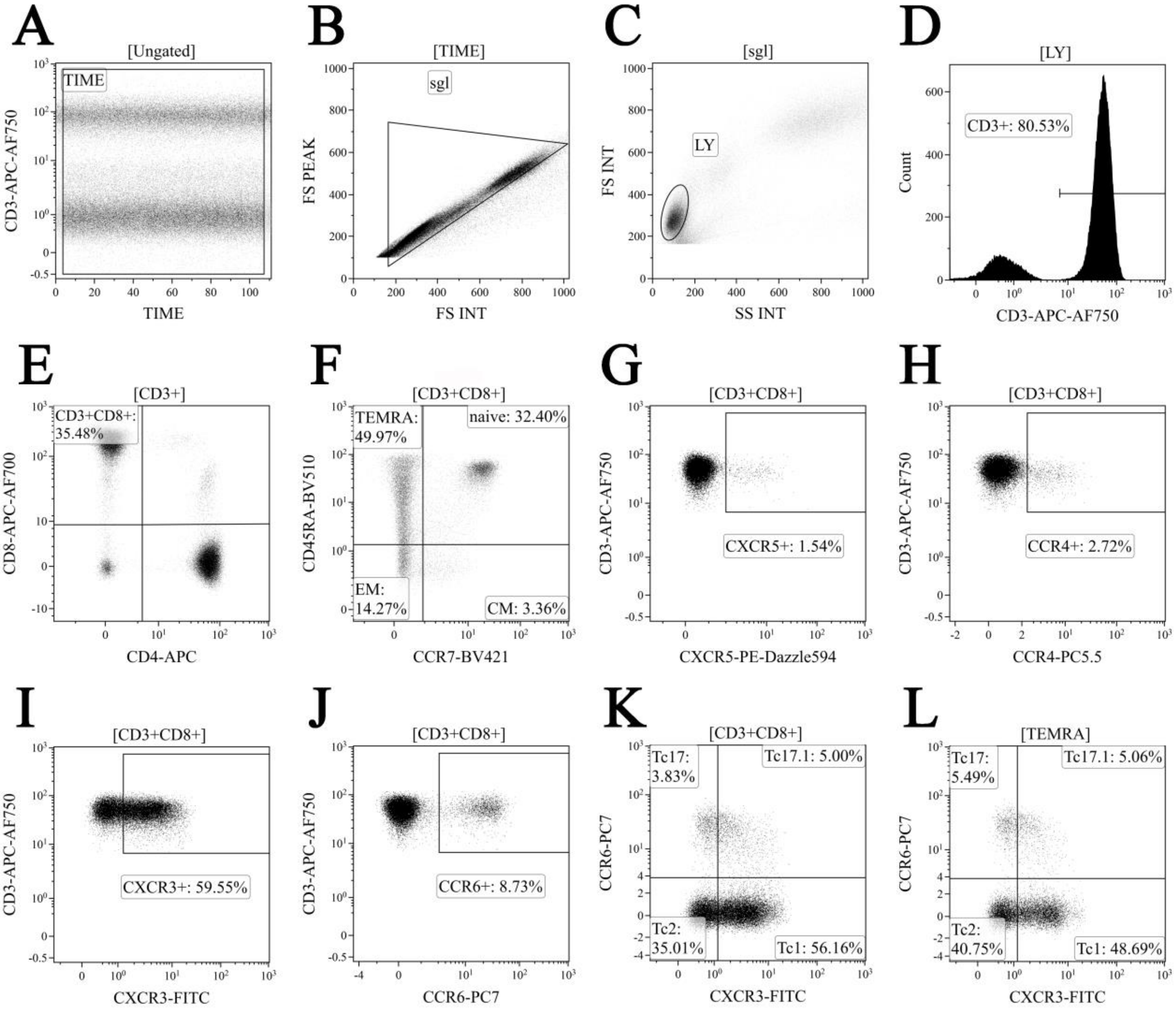

Supplementary Figure S2. Gating and analysis strategy for 'polarized' CD8+ T cell subsets immunophenotyping using flow cytometry. Dot plot (A) – artifact exclusion included time gating; dot plot (B) – doublets exclusion from the analysis using the ratio between integral and peak forward scatter signals; dot plot (C) – total lymphocyte subset purification based on side scatter and forward scatter; dot plot (D) – total T cell subset gaiting based on CD3 expression; dot plot (E) – detection of CD8+ T cells within total CD3+ T cell subset; dot plot (F) – identification of four main CD8+ T cell maturation subsets: 'naïve', central memory (CM), effector memory (EM), and TEMRA CD8+ T cells; dot plots (G–L) – examples of CXCR5, CCR4, CXCR3, and CCR6 expression by total CD8+ T cells; dot plots – examples of Tc1 (CCR6−CXCR3+), Tc2 (CCR6−CXCR3−), Tc17 (CCR6+CXCR3−), and double-positive Tc17.1 (CCR6+CXCR3+) detection within total CD8+ T cell subset and TEMRA CD8+ T cell, respectively.

Supplementary Table S3. **A**bsolute numbers CD57-expressing cells in diverse CD8+ T cell subsets in patients with pulmonary tuberculosis.

**CD8+ T cell subset**	**Phenotype**	**TB group (n=32)** (%, Med Q25; Q75)	**Healthy control (n=31)** (%, Med Q25; Q75)	**Significant Differences**
**«Naïve»**	CD45RA+CD62L+	1 (1; 1)	1 (1; 2)	0.296
**CM**	CD45RA–CD62L+	1 (1; 2)	2 (1; 3)	0.058
**EM:**	CD45RA–CD62L–	63 (26; 113)	69 (37; 121)	0.690
**EM1**	CD27+CD28+	3 (2; 6)	4 (2; 9)	0.145
**EM2**	CD27+CD28–	9 (4; 18)	10 (6; 15)	0.794
**EM3**	CD27–CD28–	37 (17; 75)	46 (16; 98)	0.934
**EM4**	CD27–CD28+	1 (1; 3)	1 (1; 2)	0.367
**TEMRA:**	CD45RA+CD62L–	65 (42; 152)	64 (41; 110)	0.527
**pE1**	CD27+CD28+	1 (1; 2)	1 (1; 2)	0.650
**pE2**	CD27+CD28–	6 (4; 13)	6 (4; 10)	0.880
**effectors**	CD27–CD28–	62 (38; 147)	55 (33; 96)	0.364

The quantitative data (the absolute numbers of CD57-expressing cells within CD8+ T cell subsets) are presented as median and quartile ranges (Med (Q25; Q75). The statistical analysis was performed with the Mann–Whitney U test.
